# Balancing trade-offs between biotic and abiotic stress responses through leaf age-dependent variation in stress hormone cross-talk

**DOI:** 10.1073/pnas.1817233116

**Published:** 2019-01-23

**Authors:** Matthias L. Berens, Katarzyna W. Wolinska, Stijn Spaepen, Jörg Ziegler, Tatsuya Nobori, Aswin Nair, Verena Krüler, Thomas M. Winkelmüller, Yiming Wang, Akira Mine, Dieter Becker, Ruben Garrido-Oter, Paul Schulze-Lefert, Kenichi Tsuda

**Affiliations:** ^a^Department of Plant-Microbe Interactions, Max Planck Institute for Plant Breeding Research, 50829 Cologne, Germany;; ^b^Department of Molecular Signal Processing, Leibnitz Institute of Plant Biochemistry, 06120 Halle, Germany;; ^c^Cluster of Excellence on Plant Sciences, Max Planck Institute for Plant Breeding Research, 50829 Cologne, Germany

**Keywords:** combined stress, phytohormone, plant fitness, microbiota, stress trade-off

## Abstract

In nature, plants must respond to multiple stresses simultaneously, which likely demands cross-talk between stress-response pathways to minimize fitness costs. Here we provide genetic evidence that biotic and abiotic stress responses are differentially prioritized in *Arabidopsis thaliana* leaves of different ages to maintain growth and reproduction under combined biotic and abiotic stresses. Abiotic stresses, such as high salinity and drought, blunted immune responses in older rosette leaves through the phytohormone abscisic acid signaling, whereas this antagonistic effect was blocked in younger rosette leaves by *PBS3*, a signaling component of the defense phytohormone salicylic acid. Plants lacking *PBS3* exhibited enhanced abiotic stress tolerance at the cost of decreased fitness under combined biotic and abiotic stresses. Together with this role, *PBS3* is also indispensable for the establishment of salt stress- and leaf age-dependent phyllosphere bacterial communities. Collectively, our work reveals a mechanism that balances trade-offs upon conflicting stresses at the organism level and identifies a genetic intersection among plant immunity, leaf microbiota, and abiotic stress tolerance.

## Significance

Plants are exposed to conflicting stresses simultaneously in nature. As stress responses are costly, plants likely coordinate these responses to minimize fitness costs. The nature and extent to which plants employ inducible mechanisms to cope with combined physical and biological stresses remains unknown. We identify a genetic mechanism by which leaves of distinct ages differentially control stress-response cross-talk. At the organism level, this mechanism balances stress-response trade-offs to maintain plant growth and reproduction during combined stresses. We also show that this leaf age-dependent stress-response prioritization influences the establishment of plant-associated leaf bacterial communities. This study illustrates the importance of active balancing of stress-response trade-offs for plant fitness maintenance and for interaction with the plant microbiota.

In nature, plants encounter and must appropriately respond to diverse stresses to survive and reproduce. Different stress signaling pathways are connected, providing regulatory potential to maximize fitness (1). For instance, plants exposed to abiotic stresses such as high salinity and drought often display reduced immune activity (2). As stress responses are costly, prioritization in stress responses would allow plants to allocate more resources to abiotic stress responses and increase plant fitness in the absence of biotic stress (3). However, this prioritization does not explain how plants maintain fitness when simultaneously exposed to biotic and abiotic stresses.

Phytohormones play critical roles in stress responses and prioritization (4). In an evolutionarily conserved mechanism, phytohormone signaling mediated by abscisic acid (ABA) promotes abiotic stress tolerance and suppresses signaling of the biotic stress-related phytohormone salicylic acid (SA). Consequently, plant immunity is lowered during abiotic stresses such as drought and salt stress (5). In *Arabidopsis thaliana*, ABA suppresses expression of the SA biosynthesis gene *SA INDUCTION DEFICIENT 2* (*SID2*) (5). ABA application causes proteasome-mediated degradation of the SA receptor NONEXPRESSOR OF PR GENE 1 (NPR1) (6). Molecular cross-talk between ABA and SA provides a plausible mechanism for prioritization of abiotic over biotic stress responses. Such a mechanism would provide plants with adaptive potential when abiotic stresses are major threats. However, it is unknown whether this hormonal cross-talk is still advantageous when plants simultaneously encounter biotic and abiotic stresses.

In natural soils, healthy plants are colonized by taxonomically structured microbial communities, collectively called the plant microbiota (7), which contribute to plant performance under adverse environmental conditions (8–11). SA modulates colonization of the *A. thaliana* root microbiota by specific bacterial taxa, although this was not accompanied by a detectable impact on host survival of the tested SA biosynthesis and signaling mutants (12, 13). In field experiments, drought induced a relative enrichment of multiple lineages of Actinobacteria and Chloroflexi in the rice root microbiota, whereas, in sorghum roots, relative enrichments of Actinobacteria and Firmicutes were observed (14–17). Whether and how cross-talk between biotic and abiotic stress response pathways impacts the assembly of the plant microbiota remains unknown.

Age and developmental stage are important factors influencing stress responses in animals and plants. For instance, as *A. thaliana* plants age, SA-mediated immunity is enhanced (18). Plants also display age-dependent responses at the organ level. Young *A. thaliana* rosette leaves exhibit greater SA accumulation and SA-mediated immunity in comparison with older rosette leaves (19). Evidence for leaf age-dependent variation also exists for abiotic stress (20, 21). Based on the optimal defense theory (ODT), in defending themselves against herbivores, plants prioritize tissues, such as young leaves, that are more valuable for the whole plant (22). However, whether leaf age-dependent variation in stress responses is a simple prioritization analogous to the ODT or an active strategy to increase plant fitness is not understood.

Here we show that biotic and abiotic stress responses are differentially prioritized in a leaf age-dependent manner in *A. thaliana* to maintain fitness under combined stresses. Abiotic stresses dampen immunity in old rosette leaves, whereas the SA signaling components *PBS3* and *NPR1* protected young rosette leaves from ABA-mediated immune suppression. *pbs3* mutant plants exhibited enhanced abiotic stress tolerance but showed compromised fitness maintenance under combined biotic and abiotic stresses. Defining a hitherto uncharacterized link between stress signaling cross-talk and microbiota structure, *PBS3* is indispensable for the proper establishment of salt stress- and leaf age-dependent leaf bacterial communities. We propose that balancing leaf-age dependent cross-talk between SA and ABA signaling is a critical determinant of plant performance during combined stresses.

## Results

### Leaf Age Controls ABA–SA Cross-Talk Independently of Vegetative Phase Change.

**Fig. 1.**
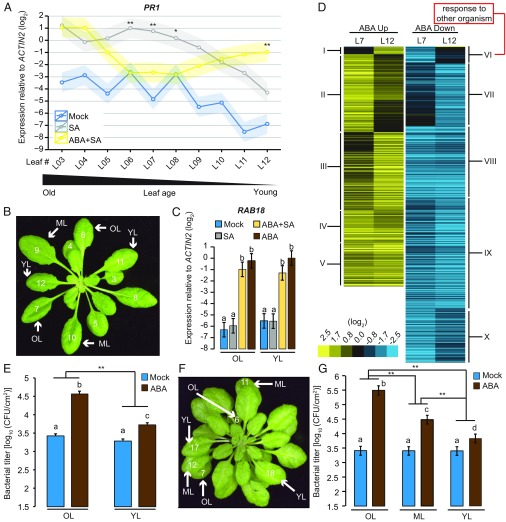
Leaf age effects of ABA on SA response and immunity. (*A*) *PR1* expression levels in leaves (from L03 to L12) of 4–5-wk-old *A. thaliana* Col-0 plants 24 h after spray with 500 µM SA, following 500 µM ABA spray pretreatment for 24 h were determined by quantitative RT-PCR. Data represent means ± SEM (shadows) calculated from three biological replicates by using a mixed linear model. Asterisks indicate significant differences between SA and combined ABA/SA treatments (*P < 0.05 and **P < 0.01, two-tailed Student’s *t* tests). (*B* and *F*) Leaf numbers in 4-wk-old Col-0 (*B*) and *35S::miR156a* (*F*) plants highlighting old (OL), middle (ML), and young leaves (YL). (*C*) *RAB18* expression levels in old and young leaves of 4–5-wk-old Col-0 plants as in *A*. Data represent means ± SEM calculated from three biological replicates by using a mixed linear model. Different letters indicate significant differences (adjusted *P* < 0.05). (*D*) Heat map showing expression patterns of the genes that show significant expression changes 48 h after ABA spray compared with mock (*q* < 0.01 and |log_2_FC| > 1) for up-regulated (yellow; 1,291 genes) or down-regulated genes (blue; 1,712 genes) in L7 or L12 of 4–5-wk-old Col-0 plants. (*E* and *G*) *Pto hrcC*^−^ (OD_600_ = 0.0002) was infiltrated into old, middle, and young leaves of 4–5-wk-old Col-0 (*E*) and *35S::miR156a* (*G*) plants 24 h after 500 µM ABA spray or mock. Bacterial growth was measured at 2 days postinoculation. Data represent means ± SEM calculated from at least three independent experiments, each with at least four biological replicates, by using a mixed linear model. Different letters indicate significant differences (adjusted *P* < 0.005; **P < 0.01, two-tailed Student’s *t* tests).

To gain insights into mechanisms underlying the cross-talk between ABA and SA signaling, we investigated the impact of ABA on the SA response in *A. thaliana*. We found that pretreatment with ABA unexpectedly blocked SA-mediated induction of the SA-response marker gene *PATHOGENESIS RELATED PROTEIN 1* (*PR1*) (5) only in a subset of rosette leaves (L06 to L08; numbers refer to the positions of rosette leaves as determined by their order of appearance), but enhanced *PR1* expression in young rosette leaves (L12; Fig. 1 *A* and *B*), indicative of a leaf age-specific effect of ABA on the SA response. In contrast, expression of the ABA-response marker gene *RESPONSE TO ABA 18* (*RAB18*) (23, 24) was independent of leaf age (Fig. 1*C* and *SI Appendix*, Fig. S1*A*).

To systematically explore the leaf age-dependency of ABA-mediated transcriptional changes, we conducted RNA sequencing (RNA-seq) experiments. Although ABA-triggered responses in old (L7) and young (L12) rosette leaves were similar overall, some gene clusters exhibited leaf age-dependent expression patterns (Fig. 1*D*, *SI Appendix*, Fig. S1 *C* and *D*, and Dataset S1). These results support our finding that expression of a subset of ABA-regulated genes is leaf age-dependent.

We next examined the physiological significance of leaf age-dependent effects of ABA on immunity. The bacterial pathogen *Pseudomonas syringae* pv. *tomato* DC3000 (*Pto*) is known to trigger ABA accumulation through the action of type III effectors (25, 26). Therefore, we reasoned that the disarmed *Pto hrcC*^−^ strain, which is unable to deliver type III effectors, can be used to detect the effect of exogenous ABA on immunity by measuring bacterial titers in leaves. We found that ABA treatment enhanced *Pto hrcC*^−^ growth more strongly in old compared with young rosette leaves (Fig. 1*E* and *SI Appendix*, Fig. S1*B*). This is consistent with an ABA-mediated suppression of the SA response in old but not in young leaves (Fig. 1*A*) and with SA-mediated immunity restricting *Pto hrcC*^−^ growth (27).

We speculated that the observed leaf age-dependent ABA effects might be linked to leaf developmental stage because the onset of detectable suppression of the SA response by ABA correlates approximately with the onset of a vegetative phase change from juvenile to adult leaves in Col-0 plants (28). To explore this possibility, we employed transgenic *A. thaliana* overexpressing the miRNA miR156a in which the expression of juvenile traits is markedly prolonged (28). However, similar to WT, we observed that ABA effects on *Pto hrcC*^−^ growth were dependent on leaf age despite the juvenile trait being manifest in younger and older leaves (Fig. 1 *F* and *G*). Thus, leaf age but not vegetative phase change likely controls ABA–SA cross-talk, with marked consequences for the SA response and bacterial resistance.

### ABA Suppresses Immunity via the ABA RESPONSIVE ELEMENT BINDING PROTEIN–SNAC-A Transcription Factor Cascade.

**Fig. 2.**
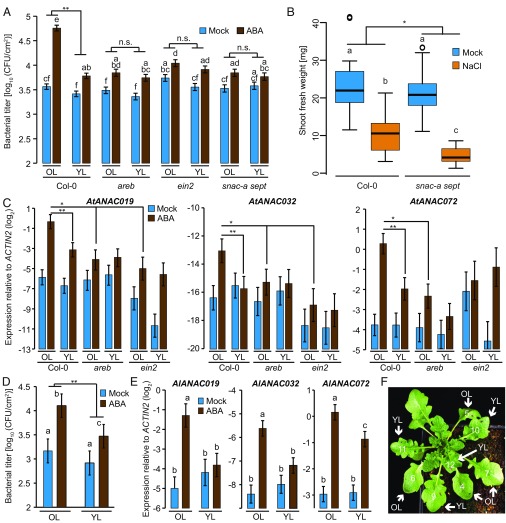
ABA triggers immune suppression in old leaves through AREB and ANAC TFs. (*A*) Old leaves (OL) and young leaves (YL) of 4–5-wk-old Col-0, *areb1 areb2 abf3* (*areb*), *ein2*, and *anac* septuple mutant (*snac-a sept*) plants were infiltrated with *Pto hrcC*^−^ (OD_600_ = 0.0002) 24 h after 500 µM ABA spray or mock. Bacterial growth was measured at 2 days postinoculation (dpi). Data represent means ± SEM calculated from three independent experiments, each with at least five biological replicates, by using a mixed linear model. Different letters indicate significant differences (adjusted *P* < 0.005). (*B*) Shoot fresh weight of Col-0 and *snac-a sept* seedlings grown on MS plates containing 100 mM NaCl or mock for 10 d. The box plots show combined data from three independent experiments, each with at least eight biological replicates. Different letters indicate significant differences (adjusted *P* < 0.05). (*C*) *AtANAC019*, *AtANAC032*, and *AtANAC072* expression levels in old and young leaves of 4–5-wk-old Col-0 and *areb1 areb2 abf3* (*areb*) plants 24 h after 500 µM ABA spray or mock were determined by quantitative RT-PCR. Data represent means ± SEM calculated from at least three biological replicates by using a mixed linear model. (*D*) *Pto hrcC*^−^ (OD_600_ = 0.0002) was infiltrated into old and young leaves of 5–6-wk-old *A. lyrata* plants 24 h after 500 µM ABA spray or mock. Bacterial growth was measured at 2 dpi. Data represent means ± SEM calculated from three independent experiments, each with at least five biological replicates, by using a mixed linear model. Different letters indicate significant differences (adjusted *P* < 0.005). (*E*) *AlANAC019*, *AlANAC032*, and *AlANAC072* expression levels in old and young leaves of 5–6-wk-old *A. lyrata* plants 24 h after 500 µM ABA spray or mock were determined by quantitative RT-PCR. Data represent means ± SEM calculated from at least three biological replicates by using a mixed linear model. Different letters indicate significant differences (adjusted *P* < 0.05). (*F*) Leaf numbers in 5–6-wk-old *A. lyrata* showing old and young leaves. (*A*–*D*) n.s., not significant (*P < 0.05 and **P < 0.01, two-tailed Student’s *t* tests).

ABA RESPONSIVE ELEMENT (ABRE) BINDING PROTEIN (AREB) transcription factors (TFs) redundantly regulate a major part of ABA-mediated transcriptional changes (24) (*SI Appendix*, Fig. S2*A*). We found that ABA-triggered suppression of immunity against *Pto hrcC*^−^ in old leaves was compromised in *areb* triple mutant plants (*areb1 areb2 abf3*) (24), indicating that ABA-triggered immune suppression requires AREB-mediated transcription. Next, we further dissected this transcriptional cascade. AREB TFs have been shown to bind to the promoters of SNAC-A TFs in vitro (29), and a subset of SNAC-A TFs, ANAC019, 055, and 072, whose gene expression is induced by ABA, are involved in the suppression of SA accumulation (30). These SNAC-A TFs redundantly control a subset of ABA responses such as *SAG26* expression and senescence, but not *RAB18* expression (23). Therefore, we tested whether SNAC-A TFs collectively regulate ABA-mediated immune suppression. Indeed, we found that ABA-mediated immune suppression in old leaves was compromised in *snac-a* septuple mutant (*snac-a sept*; *anac019 anac055 anac072/rd26 anac002/ataf1 anac081/ataf2 anac102 anac032*) (23) plants (Fig. 2*A*).

Plants overexpressing *ANAC002*, *019*, *055*, or *072* show enhanced abiotic stress tolerance (3, 31). Conversely, we found that salt tolerance was impaired in s*nac-a sept* plants (Fig. 2*B* and *SI Appendix*, Fig. S2*B*). Expression of *ANAC019*, *ANAC032*, and *ANAC072* after ABA treatment was higher in old than in young rosette leaves in an *AREB*-dependent manner (Fig. 2*C*), suggesting that this *ANAC* induction is under control of the AREBs. It has been shown that *SNAC-A* induction by ABA also requires *ETHYLENE INSENSITIVE2* (*EIN2*) (23), a key component of ethylene signaling (32). Accordingly, we found that ABA-triggered leaf age-dependent expression of *SNAC-As* and suppression of immunity against *Pto hrcC*^−^ were impaired in *ein2* plants (Fig. 2 *A* and *C*), whereas *RAB18* induction remained intact (*SI Appendix*, Fig. S2*A*). This corroborates the conclusion that these SNAC-A TFs play an important role in ABA-mediated suppression of immunity in old leaves. Collectively, these results suggest that *snac-a sept* plants exhibit an altered balance between biotic and abiotic stress responses.

To explore the evolutionary conservation of the leaf age-dependent effect of ABA on immunity, we investigated *Arabidopsis lyrata*, a close relative of *A. thaliana*. We found that ABA suppresses immunity against *Pto hrcC*^−^ and induces *SNAC-A* expression in a leaf age-dependent manner as in *A. thaliana* Col-0 plants (Fig. 2 *D*–*F*) (33).

### *PBS3* Protects Young Leaves from ABA-Triggered Immune Suppression.

**Fig. 3.**
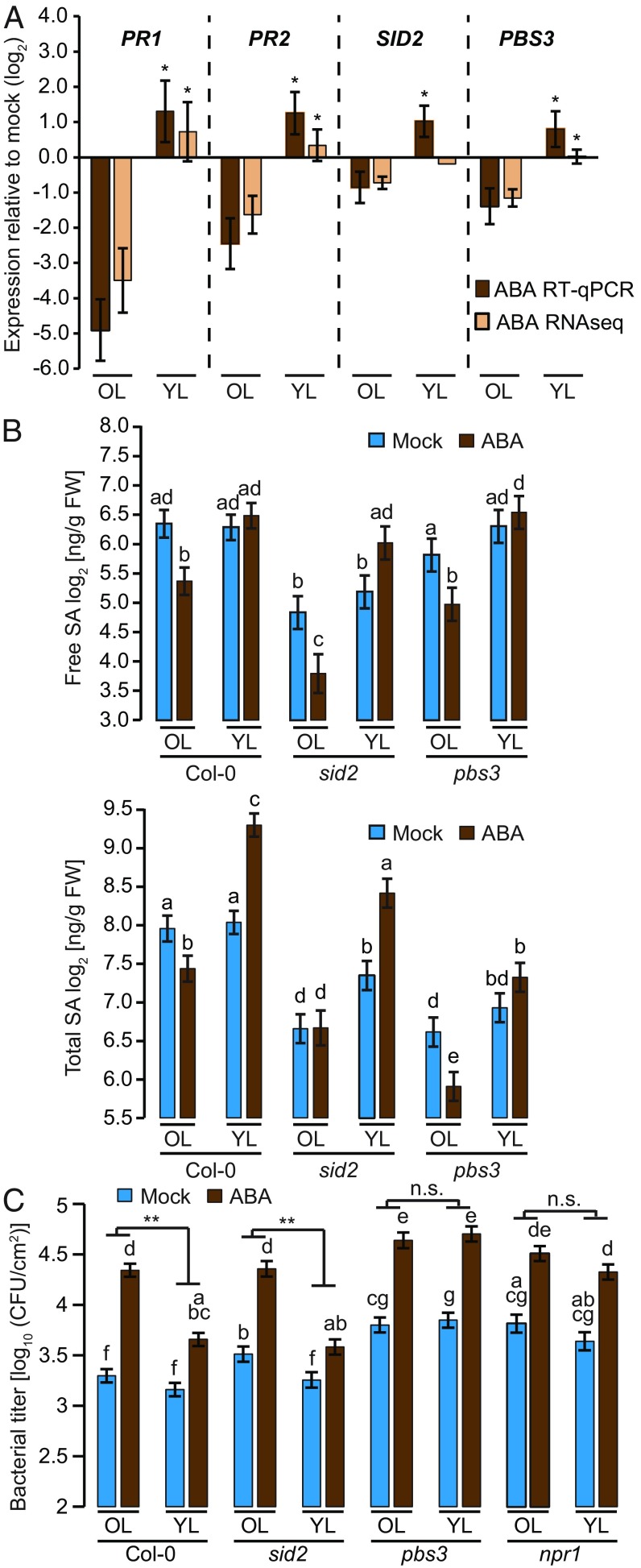
*PBS3* protects young leaves from ABA-triggered immune suppression. (*A*) The expression changes of *PR1*, *PR2*, *SID2*, and *PBS3* in old leaves (OL) and young leaves (YL) of 4–5-wk-old Col-0 plants 48 h after 500 µM ABA spray compared with mock in RNA-seq and quantitative RT-PCR. Data represent means ± SEM of three biological replicates. Asterisks indicate significant differences in young compared with old leaves (*P < 0.05, two-tailed Student’s *t* tests). (*B*) Free and total SA amounts in old and young leaves of 4–5-wk-old Col-0, *sid2*, and *pbs3* plants 48 h after spray with 500 µM ABA or mock. Data represent means ± SEM calculated from three biological replicates by using a mixed linear model. Different letters indicate significant differences (adjusted *P* < 0.05). (*C*) Old and young leaves of 4–5-wk-old Col-0, *sid2*, *pbs3*, and *npr1* plants were infiltrated with *Pto hrcC*^−^ (OD_600_ = 0.0002) 24 h after 500 µM ABA spray or mock. Bacterial growth was measured at 2 days postinoculation. Data represent means ± SEM calculated from three independent experiments, each with at least five biological replicates, by using a mixed linear model. Different letters indicate significant differences (adjusted *P* < 0.005; *P < 0.05 and **P < 0.01, two-tailed Student’s *t* tests). n.s., not significant.

Our RNA-seq experiments described here earlier revealed that ABA mediates a strong suppression of genes enriched for the Gene Ontology term “response to other organism” specifically in old leaves (L7; cluster VI; Fig. 1*D*). Cluster VI included *PBS3*, encoding an acyl-adenylate/thioester-forming enzyme, which is known to be important for SA accumulation and signaling and for immunity against biotrophic pathogens. Cluster VI also included the SA response markers *PR1* and *PR2* (34), suggesting that SA signaling components are specifically suppressed in old leaves. Therefore, although it is not in cluster VI, we also included *SID2* for further analysis because it encodes a key enzyme for pathogen-induced SA biosynthesis (35). ABA reduced the expression of all tested genes in old leaves but did not in young leaves (Fig. 3*A*). Next, we determined free and total SA levels after ABA application in Col-0, *sid2*, and *pbs3* plants and found that ABA had contrasting effects on SA levels in old and young Col-0 leaves (Fig. 3*B*). Strikingly, the increased total SA, mediated by ABA treatment in young leaves of Col-0, was retained in *sid2* but abolished in *pbs3* plants (Fig. 3*B*). Given that PBS3 has been proposed to protect SA from degradation (36), we conclude that the increase in total SA elicited by ABA in young leaves may be caused by reduced SA degradation rather than increased SA biosynthesis via SID2.

Notably, we found that young leaves of *pbs3* plants are vulnerable to ABA-mediated suppression of immunity against *Pto hrcC*^−^, whereas *sid2* plants exhibited a WT-like phenotype (Fig. 3*C*). In addition, we found that the enhanced SA-induced *PR1* expression by ABA in young leaves is compromised in *pbs3* plants whereas ABA-induced *RAB18* expression remains intact (*SI Appendix*, Fig. S3 *A* and *B*). Finally, increased free and total SA accumulation was impaired in *pbs3* leaves upon treatment with flg22, a peptide epitope derived from bacterial flagellin that stimulates SA accumulation (27) (*SI Appendix*, Fig. S3 *C* and *D*). Thus, *PBS3* is required for immunity-triggered SA accumulation. In young leaves, however, increased total SA accumulation and SA response upon ABA treatment might be important to protect young leaves from ABA-mediated immune suppression, which is impaired in *pbs3* plants.

Given that *NPR1*, encoding the SA receptor (37, 38), is required for the majority of the SA response (37) (*SI Appendix*, Fig. S3*B*), we also included *npr1* plants in our analysis. We found that, similar to *pbs3*, young leaves of *npr1* plants are not protected against ABA-mediated immune suppression (Fig. 3*C*). These and previous results might suggest that *NPR1* is required for *PBS3* function or vice versa (39). ABA promotes NPR1 degradation, which correlated with reduced *PR1* expression, whereas SA antagonizes this (6). Together, NPR1 might be protected from ABA-mediated degradation by higher SA levels in young leaves. Our finding that expression of *SNRK2.8*, an important regulatory interactor of NPR1 (40), is strongly suppressed by ABA in only old leaves supports this hypothesis (*SI Appendix*, Fig. S1 *C* and *D*).

### *PBS3* Regulates the Trade-Off Between Biotic and Abiotic Stress Responses.

**Fig. 4.**
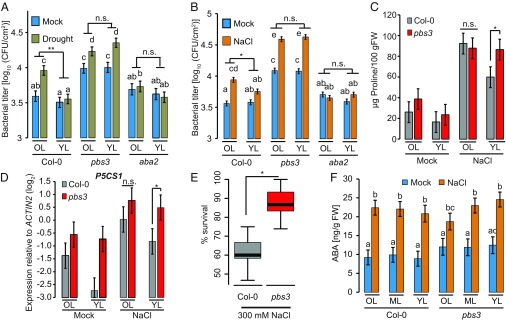
Impact of *PBS3* on leaf age-dependent stress-response trade-offs. (*A* and *B*) Old leaves (OL) and young leaves (YL) of 4–5-wk-old Col-0, *pbs3*, and *aba2* plants were infiltrated with *Pto hrcC*^−^ (OD_600_ = 0.0002) 2–3 wk after transfer to drought or well-watered conditions (mock) at 2-wk-old stage (*A*) or 2 d after water or 75 mM NaCl treatment (*B*). Bacterial growth was measured at 3 days postinoculation (dpi) (*A*) or 2 dpi (*B*). Data represent means ± SEM calculated from three independent experiments, each with at least five biological replicates, by using a mixed linear model. Different letters indicate significant differences (adjusted *P* < 0.01). (*C* and *D*) Proline (*C*) or *P5CS1* expression levels (*D*) in old and young leaves of 4–5-wk-old Col-0 and *pbs3* plants 6 d after 100 mM NaCl or mock treatment. Data represent means ± SEM calculated from three biological replicates by using a mixed linear model. (*E*) Survival rate of Col-0 and *pbs3* plants after salinity stress recovery. Two-week-old plants were watered with 300 mM NaCl for 2 wk followed by recovery with water for another 1 wk. Data consist of three independent experiments, each with at least 35 plants per genotype. (*F*) The ABA levels in old, middle, and young leaves of 4–5-wk-old Col-0 and *pbs3* plants 6 h after 100 mM NaCl or mock soil drench treatment. Data represent means ± SEM calculated from at least three biological replicates by using a mixed linear model. Different letters indicate significant differences (adjusted *P* < 0.05). (*A*–*E*) *P < 0.05 and **P < 0.01, two-tailed Student’s *t* tests; n.s., not significant.

Abiotic stresses such as salinity and drought activate ABA biosynthesis (41). To test whether endogenous ABA accumulation induced by abiotic stresses triggers leaf age-dependent immune suppression, we measured *Pto hrcC*^−^ growth in *A. thaliana* plants after salt or drought treatment. To avoid the known pleiotropic effects of SA hyperaccumulation in *npr1* on plant performance (42, 43), we focused on *pbs3* plants. Similar to the ABA treatment described here earlier, young leaves of Col-0 plants were protected from salt and drought stress-triggered immune suppression (Fig. 4 *A* and *B*). Abiotic stress-triggered suppression of immunity against *Pto hrcC*^−^ in old leaves was not seen in *aba2*, which is impaired in ABA biosynthesis (44) (Fig. 4 *A* and *B*), indicating that abiotic stress-induced immune suppression is dependent on endogenous ABA and/or derived metabolites from ABA (45). In contrast to WT Col-0, young leaves of *pbs3* plants were not protected from the abiotic stress-triggered immune suppression (Fig. 4 *A* and *B*). Thus, the protective role of *PBS3* in young leaves is physiologically relevant.

Considering the extensive cross-talk between biotic and abiotic stress responses in both directions (2), we hypothesized that young leaves of *pbs3* are hyperresponsive to abiotic stress compared with those of WT. To measure a leaf-specific abiotic stress response output, we determined the accumulation of proline, which serves as a cellular osmoprotectant (46). Upon salt stress, proline levels and expression of *P5CS1*, which encodes an enzyme that catalyzes the rate-limiting step in proline biosynthesis (46), were significantly higher only in young leaves of *pbs3* compared with Col-0 (Fig. 4 *C* and *D*). Together with the compromised immune phenotype, it appears that, in *pbs3* plants, the balance between biotic and abiotic stress responses is shifted toward abiotic stress tolerance. In agreement with this, under severe salt stress (300 mM NaCl), which can be lethal to *A. thaliana* plants, the survival rate of *pbs3* was higher than that of Col-0 plants (Fig. 4*E*).

Interestingly, Col-0 and *pbs3* plants accumulated comparable levels of basal and salt stress-induced ABA in leaves of different ages (Fig. 4*F*). We also determined the levels of other phytohormones, namely auxin (indole-3-acetic acid; IAA), jasmonic acid and its precursor 12-oxo-phytodienoic acid, SA, and the ethylene precursor 1-aminocyclopropane-1-carboxylic acid (ACC) (47) in Col-0 and *pbs3* leaves of different ages upon salt stress or mock treatment. Although these hormone levels were mostly unaffected by genotype or salt stress, levels of IAA and ACC were dependent on leaf age (*SI Appendix*, Fig. S4), which may influence the leaf age-dependent stress cross-talk. In summary, these results suggest that, during combined stress, *PBS3* lowers the abiotic stress response and enhances immunity in young leaves.

### *PBS3* Is Required for the Maintenance of Plant Growth and Reproduction Under Combined Stress.

**Fig. 5.**
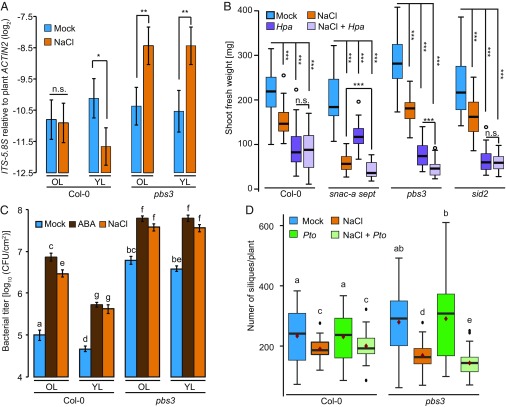
Leaf age-dependent variation in biotic and abiotic stress cross-talk contributes to plant fitness-related traits under combined stresses. (*A*) *Hpa* growth 8 d after inoculation in old leaves (OL) and young leaves (YL) of 4–5-wk-old Col-0 and *pbs3* plants following 75 mM NaCl or water (Mock) soil drench treatment for 2 d. Data are means ± SEM calculated from at least three biological replicates by using a mixed linear model. (*B*) Shoot fresh weight of Col-0, *snac-a sept*, *pbs3*, and *sid2* plants challenged with mock, NaCl, *Hpa*, or both NaCl and *Hpa* (*Methods*). The box plots show combined data from at least three independent experiments for Col-0, *pbs3*, and *sid2* and two independent experiments for *snac-a sept* mutant plants, each with at least eight biological replicates. (*A* and *B*) *P < 0.05, **P < 0.01, and ***P < 0.001, two-tailed Student’s *t* tests; n.s., not significant. (*C*) Old and young leaves of 4–5-wk-old Col-0 and *pbs3* plants were infiltrated with *Pto cor*^−^ (OD_600_ = 0.0002) 1 d after water, 500 µM ABA, or 100 mM NaCl treatment. Bacterial growth was measured at 2 days postinoculation. Data represent means ± SEM calculated from three independent experiments, each with at least five biological replicates, by using a mixed linear model. Different letters indicate significant differences (adjusted *P* < 0.005). (*D*) The number of siliques in Col-0 and *pbs3* plants after water (Mock), 50 mM NaCl (NaCl), *Pto cor*^−^ (*Pto*), or both NaCl and *Pto*. The box plots show combined data from three independent experiments, each with at least 10 biological replicates. Statistical analysis was performed by using log-transformed silique numbers. Different letters indicate significant differences (adjusted *P* < 0.05).

We employed two experimental systems to test whether the altered balance of leaf age-dependent stress cross-talk in *pbs3* plants affects plant fitness-related traits during combined stresses. In the first system, we combined salt stress with infection with the obligate biotrophic oomycete pathogen *Hyaloperonospora arabidopsidis* (*Hpa*), whose growth is sensitive to SA-mediated immunity (48). Considering that many plant pathogens, including *Hpa*, require high humidity for successful infection, co-occurrence of drought stress with pathogen infection is unlikely to be common. Therefore, we used mild salt stress as an abiotic stress factor, which can co-occur with pathogen infection (49). Salt pretreatment reduced oomycete biomass in the young leaves of Col-0 WT, whereas, in *pbs3* plants, salt stress promoted *Hpa* growth in old and young leaves (Fig. 5*A*). We included *snac-a sept* plants in the plant performance assay because, in this genotype, the balanced trade-offs of abiotic and biotic stress responses were shifted in the opposite direction compared with *pbs3* plants (Figs. 2 *A* and *B*, 3*C*, and 4). Furthermore, we included *sid2* plants because of their deficiency in SA biosynthesis but indistinguishable leaf age-dependent stress cross-talk vs. Col-0 plants (Fig. 3*C*). We found that single salt and *Hpa* stress reduced shoot fresh weight of all tested genotypes, with *Hpa* infection having greater negative consequences on growth of Col-0, *pbs3*, and *sid2*, except *snac-a sept* plants, whose growth suffered more severely from salt stress (Fig. 5*B*). This *snac-a sept* plant phenotype is consistent with our observation that *snac-a sept* plants exhibited lowered tolerance to mild salt stress in a germfree environment (Fig. 2*B*).

Interestingly, the effects of combined stress on plant growth were similar to those of *Hpa* single stress in WT and *sid2* plants, which exhibited leaf age-dependent stress cross-talk (Figs. 3*C* and 5*B*). In contrast, combined stress had more severe consequences on plant growth than the single *Hpa* and salt stress in *pbs3* and *snac-a sept* plants, respectively (Fig. 5*B*). Thus, these results suggest that a loss in leaf age-dependent stress-response cross-talk is associated with a plant growth penalty during combined stress conditions.

In the second assay, we used salt stress and infection with *Pto cor*^−^ as an additional biotic and abiotic stress combination. The *Pto cor*^−^ strain lacks the phytotoxin coronatine that can suppress immune responses such as SA accumulation and MAPK activation (30, 50) but is more virulent compared with *Pto hrcC*^−^ (Figs. 1*E* and 5*C*). Similar to *Pto hrcC*^−^ infection, immune suppression by ABA treatment and salt stress was leaf age-dependent in Col-0 but independent of leaf age in *pbs3* plants (Fig. 5*C*). To evaluate whether *A. thaliana* reproduction was affected by abiotic and biotic response cross-talk, we measured the number of siliques under single salt or *Pto cor*^−^ stress or in combination in Col-0 and *pbs3* plants. Under our experimental conditions, *Pto cor*^−^ infection alone did not affect silique numbers of Col-0 and *pbs3* plants, but moderate salt stress resulted in a reproductive penalty (Fig. 5*D*). In Col-0 plants, the effect of combined stress on silique numbers was similar to that of salt stress alone, whereas the negative effect of combined stress exceeded the single salt stress in *pbs3* plants (Fig. 5*D*). Together, these findings indicate that *PBS3*, which is necessary for balancing leaf age-dependent biotic and abiotic stress responses based on leaf age, is required for the maintenance of growth and reproduction under combined biotic and abiotic stress.

### *PBS3* Coordinates Leaf Age- and Salt Stress-Dependent Phyllosphere Microbiota Assembly.

**Fig. 6.**
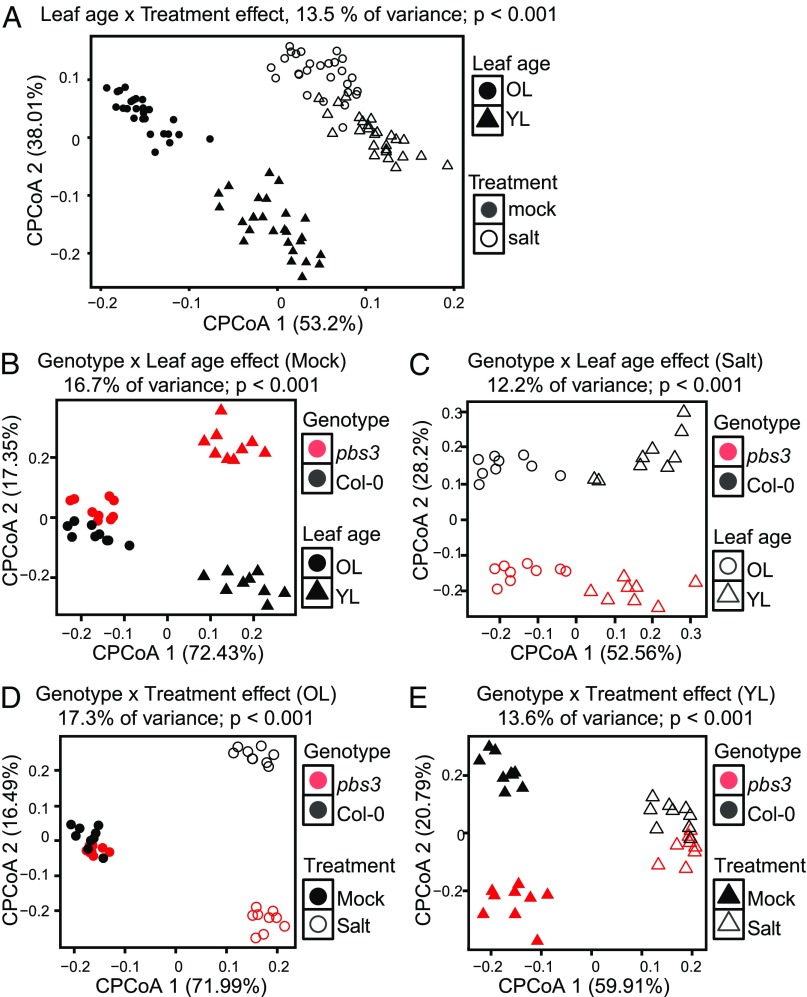
*PBS3* shapes the leaf age- and salt stress-dependent assembly of leaf bacterial communities. (*A*–*E*) Canonical analysis of principle coordinates of bacterial β-diversity Bray–Curtis distances based on bacterial 16S rRNA profiling of leaf bacterial communities in WT Col-0, *pbs3*, and *aba2* (*A*) or Col-0 and *pbs3* plants (*B*–*E*). Plants were grown in natural Cologne soil treated with water (mock) or 75 mM NaCl (salt) for 6 wk. Constrained analysis was performed for leaf age × treatment effect (*A*), genotype × leaf age effect under mock (*B*) or salt stress (*C*), and genotype × treatment effect in old leaves (OL; *D*) or young leaves (YL; *E*).

To test the impact of leaf age-dependent variation in cross-talk between ABA and SA signaling on biotic components beyond pathogens, we compared the composition of leaf-associated bacterial communities under abiotic stress or control in old and young rosette leaves. We used surface-sterilized *A. thaliana* seeds sown in a natural loamy soil with a characterized soil biome (7). Exposure of the plants to chronic salt stress resulted in decreases in plant fresh weight (*SI Appendix*, Fig. S5*A*). We applied bacterial 16S rRNA gene amplicon sequencing for quantitative bacterial community profiling and found upon principal coordinate analysis (PCoA) that salt treatment and leaf age were major factors in determining leaf microbiota composition (explaining 13.5% of community variation; Fig. 6*A* and *SI Appendix*, Fig. S5*B*). In addition, compared with Col-0, the leaves of *aba2* plants were inhabited by more distinct bacterial communities under salt stress than the control condition (*SI Appendix*, Fig. S5 *C* and *D*), indicating that the salt stress-induced shift in the leaf-associated bacterial community is, at least in part, controlled by plant ABA signaling.

Notably, young leaves of *pbs3* and WT Col-0 plants hosted distinct bacterial communities in comparison with their old leaves in the control condition (16.7% of variation; Fig. 6*B*). Under salt stress, old and young leaves of these two genotypes all harbored distinct bacterial communities (12.2% of variation; Fig. 6*C*). To directly visualize the effects of salt stress on bacterial community composition in these genotypes, we also performed PCoA analysis for young and old leaves separately. This refined analysis revealed that, under salt stress, old leaves of *pbs3* and Col-0 plants hosted distinct bacterial communities in comparison with the control condition (17.3% of variation; Fig. 6*D*). In contrast, bacterial communities in the young leaves of *pbs3* and Col-0 plants were more distinct under control conditions than under salt stress (13.6% of variation; Fig. 6*E*). Together, these results show that *pbs3* and Col-0 plants assemble distinct leaf age-dependent bacterial communities and that salt stress alters the community profiles in these two genotypes in a distinctive manner.

**Fig. 7.**
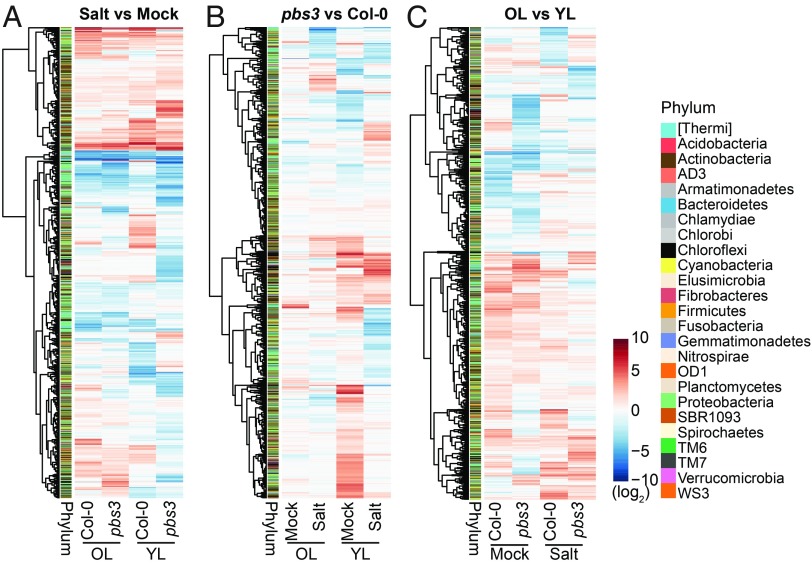
*PBS3*, salt stress, and leaf age determine relative bacterial abundances. (*A*) Heat map displaying log_2_ fold changes of relative abundances in bacterial OTUs under salt stress compared with mock in old leaves (OL) and young leaves (YL) of WT Col-0 and *pbs3* plants. (*B*) Heat map displaying log_2_ fold changes of relative abundance for bacterial OTUs in old and young leaves of *pbs3* compared with Col-0 plants under mock or salt conditions. (*C*) Heat map displaying log_2_ fold changes of relative abundance for bacterial OTUs in old leaves compared with young leaves of Col-0 and *pbs3* plants under mock or salt conditions. (*A*–*C*) Plants were grown in natural Cologne soil treated with water (Mock) or 75 mM NaCl (Salt) for 6 wk. The log_2_ fold changes were subjected to hierarchical clustering. The phylum to which each OTU belongs is indicated by the colored bar.

Salt stress altered the relative abundance of a broad range of bacterial operational taxonomic units (OTUs) belonging to different phylogenetic lineages rather than a specific taxonomic group in plant leaves and unplanted soil (Fig. 7*A*, *SI Appendix*, Figs. S5*B* and S6*A*, and Dataset S2). Shannon diversity analysis suggests that the different plant genotypes affected bacterial community profiles rather than overall bacterial richness (*SI Appendix*, Fig. S5*E*). Analysis at the level of OTUs revealed that *PBS3* affects relative abundances of a wide range of leaf-associated bacteria including Actinobacteria, Firmicutes, and Proteobacteria (Fig. 7*B* and *SI Appendix*, Fig. S6*B*). Similarly, the distinctive leaf age-dependent community shifts seen in *pbs3* plants influence a wide range of leaf-associated bacterial OTUs belonging to different phyla (Fig. 7*C* and *SI Appendix*, Fig. S6*C*). Thus, *PBS3*, salt stress, and leaf age broadly affected leaf-associated bacterial community assemblages. Together, these findings extend the physiological significance of variation in leaf age-dependent biotic and abiotic stress cross-talk to the leaf microbiota.

## Discussion

We have unveiled a genetically controlled mechanism by which *A. thaliana* plants balance trade-offs between conflicting biotic and abiotic stress responses by integrating these differently in young and old leaves. Moreover, we find that leaf age-dependent preference of stress responses balances trade-offs to increase plant growth and reproduction during combined stress. Thus, our findings define a plant strategy to maintain fitness in nature, where plants are often exposed to multiple stresses simultaneously (51), and demonstrate the physiological significance of stress-hormone cross-talk at the organismal level.

Young leaves serve as a better energy source compared with old leaves because cellular components such as the photosynthesis apparatus are more intact (52–55). The ODT explains why plants prioritize young leaves over old leaves for defense against insect herbivores by postulating that young leaves constitute a higher value for the whole plant, where value is correlated with the cost of having that tissue removed (22). Our study shows that young leaves exhibit higher biotic stress responses but lower abiotic stress responses compared with old leaves. Thus, our work suggests that young leaves are not simply protected from stresses because of their higher value in a manner similar to the ODT, but rather that plants actively balance the trade-off between biotic and abiotic stress responses through leaf age-dependent variation in stress hormone cross-talk. *pbs3* and *snac-a sept* plants, in which this balancing trade-off mechanism was absent, exhibit fitness penalties during combined biotic and abiotic stress conditions. In nature, actively maintaining fitness during combined stresses might be crucial for plant reproduction, and the balancing trade-off mechanism adds another dimension to our understanding of how plants cope with complex and fluctuating environments.

Our genetic analysis revealed that leaf age-dependent stress response prioritization during combined stress is controlled by *PBS3* and *NPR1*, components of SA signaling. However, SA biosynthesis via *SID2* in the isochorismate pathway was not required. Hence, this mechanism is distinct from plant age-dependent control of plant immunity as described during age-related resistance, which is fully dependent on *SID2* in *A. thaliana* (18). Another study reported that expression of *ENHANCED DISEASE SUSCEPTIBILITY5* (*EDS5*), required for SA accumulation, shows leaf age-dependency in *A. thaliana* (56). However, *EDS5* expression is higher in older compared with younger leaves. Therefore, this *EDS5* expression pattern does not explain why young leaves are protected from ABA-triggered immune suppression. Thus, the leaf age-dependent trade-off between biotic and abiotic stress responses during combined stress is regulated by a mechanism distinct from the previously described age-dependent variation in stress responses. Our findings also indicate that the function of SA is not limited to plant–microbe interactions, but has wider implications for plant fitness maintenance under combined stress. Consistent with this, *A. thaliana npr1* mutants exhibited reduced fitness in the field but not under controlled standard conditions (57).

Our data show that salt stress, leaf age, plant ABA biosynthesis, and *PBS3* influence the structure of leaf-associated bacterial communities. Factors determining microbiota structure and contributions of plant commensals to plant health and fitness are beginning to be defined (13). Our findings demonstrate that leaf age-dependent variation in biotic and abiotic stress cross-talk is not limited to interactions with microbial pathogens, but also influences associations with resident leaf commensals. In the context of the observed differential biotic and abiotic stress response prioritization in younger and older leaves, this raises the intriguing possibility that the corresponding distinctive leaf-resident bacterial communities are adapted to contribute preferentially to biotic and abiotic stress tolerance, respectively. Irrespective of this, our work identifies a leaf age-dependent genetic intersection among immunity, the leaf-associated bacterial microbiota, and abiotic stress tolerance, which might determine plant fitness in natural environments.

## Materials and Methods

Plants were grown in a chamber at 22 °C with 60% relative humidity and a 10-h light period for 4 wk before transfer to another chamber at 22 °C with 60% relative humidity and a 12-h light period before treatments. All *A. thaliana* plants used were in the Col-0 accession background. The details and procedures of plant materials and growth conditions, bacterial infection, *Hpa* infection, performance assay, quantitative PCR, RNA-seq, SA measurements, proline quantification, quantification of multiple phytohormones, bacterial 16S rRNA gene profiling, and statistical analysis, as well as the gene accession numbers used in this study, are provided in *SI Appendix*, *SI Materials and Methods*.

## Supplementary Material

Supplementary File

Supplementary File

Supplementary File

Supplementary File
