# Supplementary material for: Balancing trade-offs between biotic and abiotic stress responses through leaf age-dependent variation in stress hormone cross-talk
Source: Proc Natl Acad Sci U S A. 2019 Jan 23;116(6):2364–73. doi: 10.1073/pnas.1817233116 (PMC6369802; doi:10.1073/pnas.1817233116)
Supplement: Supplementary File [file pnas.1817233116.sapp.pdf]

SI Appendix

SI Materials and Methods

Plant materials and growth conditions

Plants were grown in a chamber at 22°C with 60% relative humidity and a 10-h light period for four weeks before transfer to another chamber at 22°C with 60% relative humidity and a 12-h light period prior to treatments. All *A. thaliana* plants used were in the Col-0 accession background. The *aba2-12*, *ein2-1*, *sid2-2*, *pbs3-1*, *npr1-1*, *areb1 areb2 abf3* (*areb*), and *anac019 anac055 anac072/rd26 anac002/ataf1 anac081/ataf2 anac102 anac032* (*snac-a sept*) mutants as well as the transgenic plant *35S::miR156a* were described previously (1-7). The MN47 accession was used for *A. lyrata* experiments (8). True rosette leaves L6 and L7 as old (OL), L9 and L10 as middle (ML), and L11 and L12 as young leaves (YL) for *A. thaliana* Col-0 and true rosette leaves L4-7 as old (OL) and L10-12 as young leaves (YL) for *A. lyrata* MN47 were used unless otherwise described.

Bacterial infection

Pto DC3000 strains were grown overnight in 5 ml liquid NYGA medium containing rifampicin (40 µg/ml) at 28°C. Before adjusting the density, bacterial cells were washed two times with water followed by centrifugation at 6,000 rpm for 2 min and re-suspension in water. For bacterial growth assays, old and young leaves of four to five-week-old plants were infiltrated with *Pto* DC3000 *hrcC*$^-$ or *Pto* DC3000 *cor*$^-$ (OD_{600} = 0.0002). To evaluate the effect of ABA pretreatment, bacterial infection was performed 24 h after ABA (Sigma A1049, 500 µM in 0.5% EtOH) or mock (0.5% EtOH) spray treatment. In experiments determining the impact of prior salt stress, bacterial infection was performed two days after 75 mM NaCl or 100 mM NaCl single soil drench treatment for *Pto* DC3000 *hrcC*$^-$ or *Pto* DC3000 *cor*$^-$ infection, respectively. For drought stress pretreatment, watering was reduced to 20 ml every three to four days at a plant age of 2.5 weeks. The frequency of watering was increased to every two days at a plant age of four weeks to prevent wilting. Bacterial growth was measured as described previously (9). Bacterial titer was assessed as the $\log_{10}$ transformed colony forming units per cm² leaf area (cfu/cm²) and the data were fit to the following linear model: $cfu_{gyre} = GY_{gy} + R_{ex}/R_{biol} + e_{gyr}$, where GY, genotype:treatment interaction and random factors; R_{ex}, experimental trial, R_{biol} biological replicate; e, residual.

***Hpa* infection**

Spore preparation and spray infection of the *Hpa* isolate Noco2 (4 x 10^4 conidiospores/ml) were performed as described previously (10). For oomycete biomass quantification, four to five-week-old Col-0 and *pbs3* plants were spray-infected two days after water or 75 mM NaCl soil drench treatment. Plants were covered with a clear lid to increase the relative humidity to 100%. Leaf materials were collected eight days after infection,

www.pnas.org/cgi/doi/10.1073/pnas.1817233116

frozen in liquid nitrogen, and stored at -80°C. Total genomic DNA was extracted and *Hpa* biomass was quantified by qPCR as described previously (11).

Performance assay

To measure performance under single salt stress, seedlings were grown vertically for seven days on ½ MS plates supplemented with 1% sucrose (0.8% agar) at 22°C with a 10-h light period. Seedlings were then transferred to new ½ MS plates containing 1% sucrose as well as 100 mM NaCl, and grown at 22°C with a 10-h light period. Shoot fresh weight was measured ten days after transfer. For survival rate, two-week-old plants were soil-drenched with 300 mM NaCl for 14 d. Then, plants were recovered by soil drenching with water and survivors were counted seven days later. Survival rate was calculated as the percentage of survivors.

Combined *Hpa* and salt stress treatments were performed with 2.5-week-old plants. Two days prior to the infection with *Hpa*, plants were soil-drenched with 100 mM NaCl. Spore preparation and spray infection with *Hpa* isolate Noco2 (4×10^4 conidiospores/ml) were performed as described previously (10). Plants were kept with a clear cover to increase the relative humidity to 100%. Seven days after *Hpa* infection, plants were transferred to another chamber at 19°C with 90% relative humidity and a 16-h light period. After that, plants were soil-drenched with 50 mM NaCl, and shoot fresh weight was quantified seven days later.

Combined *Pto* DC3000 *cor-* and salt stress treatments were performed with four to five-week-old-plants. Plants were grown in pots that were covered with a mesh to enable infection via vacuum infiltration. Two days prior to bacterial infection, plants were transferred to a 16-h light period to induce flowering and soil-drenched with 50 mM NaCl. Vacuum infiltration (OD_{600} = 0.0002) was performed as described previously (12). Twenty days later, the frequency of watering with water or NaCl was reduced to induce fruit maturation and the numbers of siliques per plant were counted ten days later.

Readout data were fitted to the following linear model: $X_{gyre} = GY_{gy} + R_{ex}/R_{biol} + e_{gyr}$, where GY, genotype:treatment interaction and random factors; R_{ex}, experimental replicate, R_{biol} biological replicate; e, residual.

Quantitative PCR

For gene expression, four to five-week-old plants were sprayed with ABA (Sigma A1049, 500 µM in 0.5% EtOH) or mock (0.5% EtOH) for 24 h or 48 h, or pre-sprayed with ABA (Sigma A1049, 500 µM in 0.5% EtOH) or mock (0.5% EtOH) for 24 h, followed by a spray with SA (500 µM) or water for 24 h. Total RNA was extracted with TriFast (peqlab, Erlangen, Germany) and cDNA was synthesized with superscript II (Life Technologies). For oomycete biomass quantification, the amount of oomycete DNA, estimated by primers against the conserved region *ITS5.8S* was normalized to the amount of plant DNA, estimated by primers against *ACTIN2*. Quantitative PCR was performed as described previously (13). The primers used in this study are presented in Dataset S3. The log_2-transformed gene expression or biomass data were normalized to *ACTIN2* and fit to the following

model: $Ct_{yre} = Y_y + R_r + e_{yr}$ or $Ct_{gyre} = GY_{gy} + R_r + e_{gyr}$, where GY, genotype:treatment interaction and random factors; R, biological replicate; e, residual.

RNA-seq

Four to five-week-old Col-0 leaves were sprayed with ABA (Sigma A1049, 500 µM, 0.5% EtOH) or mock (0.5% EtOH) for 48 h. Total RNA was extracted with the TRIzol reagent (Invitrogen). Extracted RNA was treated with DNase I (Roche) and purified using the RNeasy MinElute Cleanup Kit (Qiagen). Library preparation after PolyA enrichment was performed with NEBNext Ultra™ Directional RNA Library Prep Kit for Illumina (New England Biolabs). Construction of libraries and sequencing were done at the Max Planck-Genome-centre Cologne (http://mpgc.mpipz.mpg.de/home/). Briefly, libraries were sequenced using HiSeq v3 chemistry on a HiSeq2500 (Illumina) system. Strand-specific sequences were mapped to the *A. thaliana* genome (TAIR 10) using Tophat2 software with default settings. Mapped reads per library were counted using HTSeq software (14). Differentially expressed genes were determined using the edgeR and limma package (15). Only genes with ten read counts per sample on average were used for analysis. Data were normalized *via* trimmed mean of M-values (TMM) normalization (16) and normalized values were log_2-transformed using the voom function (17). After fitting a linear model containing the parameters treatment, leaf age and replicate, differentially expressed genes were selected based on a log_2 fold change > 1 and q-value < 0.01. q-values were obtained by the qvalue R package (18). Uncentered correlation clustering was done in Cluster3.0 and visualized with Java TreeView (19). The RNA-seq data used in this study were deposited in the National Center for Biotechnology Information Gene Expression Omnibus database (accession no. GSE114645).

SA measurements

Leaves of four to five-week-old plants were sprayed with ABA (Sigma A1049, 500 µM in 0.5% EtOH) or mock (0.5% EtOH) and samples were harvested 48 h later. For flg22-triggered SA accumulation, leaves were infiltrated with 10 µM flg22 and samples were harvested 9 h later. Samples were stored at -80°C before analysis. SA extraction and quantification were performed as described previously (20). The following model was fitted to metabolic data: $X_{gyr} = GY_{gy} + R_r + e_{gyr}$, where GY, genotype:treatment interaction and random factors; R, biological replicate; e, residual.

Proline quantification

For quantification of salt stress-induced proline, four to five-week-old plants were soil-drenched with 100 mM NaCl, and leaves were harvested after five days of stress. Proline was extracted and quantified as described previously (21). The following model was fitted to metabolic data: $X_{gyr} = GY_{gy} + R_r + e_{gyr}$, where GY, genotype:treatment interaction and random factors, R, biological replicate; e, residual.

Parallel quantification of multiple phytohormones

Multiple phytohormones were measured in the leaves of four to five-week-old plants 6 h, 12 h, 24 h, and 72 h after 100 mM NaCl soil drench treatment. Parallel quantification of stress-induced ABA, ACC, IAA, JA, OPDA, and SA levels was done as described previously (22). The following models were fitted to metabolic data: $X_{gyr} = GY_{gy} + R_r + e_{gyr}$ (Fig. 4f), $Ct_{gytr} = GYT_{gyt} + R_r + e_{ytr}$ (Supplementary Fig. 6), where GY, genotype:treatment interaction and random factors; GYT, genotype:treatment:time interaction and random factors, R, biological replicate; e, residual.

Bacterial 16S rRNA gene profiling

Plants were grown in natural soil originating from Cologne and were watered from the start of the experiment with either water or salt solution (50 mM or 75 mM NaCl depending on the treatment) when necessary. Seeds of Col-0, *pbs3-1*, and *aba2-12* were surface-sterilized with NaClO and 0.1% Triton X100. Plants were placed in a greenhouse under short day conditions of an 8-h of light period. After one week, extra seedlings from both experiments were removed and only four plants per pot were left to continue growing. Leaves and unplanted soil samples were harvested after six weeks of growth. Leaf samples consisted of four to eight leaves (two leaves per plant) originating from two to four plants per pot. Leaves from the bottom part of the rosette were harvested as "old" and the youngest fully developed leaves were harvested as "young". The unplanted soil sample was taken from the middle of the pot, approximately 2 cm below the soil surface. Both leaf and soil samples were frozen in liquid nitrogen directly after harvesting. All harvested samples were stored at -80°C until further processing. DNA for bacterial 16S rRNA gene profiling was prepared as described previously (23), with a few minor changes. DNA was extracted with the FastDNA Spin Kit for Soil (MP Biomedicals) following the manufacturer's protocol with modifications: incubation from step 6 was omitted and in the final step the DNA was eluted with 50 µl of nuclease-free water (Qiagen). DNA concentration was measured with a Nanodrop™. The final elongation step of the PCR reaction was 10 min and the amplicon library contained 5 ng of DNA per sample. Quality-controlled reads were mapped as described before (24). Shannon index of α-diversity, Principal Coordinates Analysis (PCoA) with Bray-Curtis distances and enrichment analysis on the single OTU level were performed in R on the normalised OTU table with previously published R scripts (24). All the plots were made in R with the use of the ggplot2 package (25). The bacterial 16S profiling data was deposited in the European Nucleotide Archive (accession no. PRJEB26793).

Statistical analysis

Statistical analysis was performed in R using mixed linear model function (lmer) from the package lme4 unless otherwise described. Standard errors were calculated from variance and covariance values after model fitting. The Benjamini-Hochberg method was applied for correction of multiple testing in figures showing all pairwise comparisons of the mean estimates.

Accession numbers

The accession numbers for the genes discussed in this article are as follows: *AtACTIN2* (AT3G18780), *AtPR1* (AT2G14610), *AtRAB18* (AT5G66400), *AtMIR156A* (AT2G25095), *AtAREB1* (AT1G45249), *AtAREB2* (AT3G19290), *AtABF3* (AT4G34000), *AtEIN2* (AT5G03280), *AtNAC019* (AT1G52890), *AtNAC055* (AT3G15500), *AtNAC072* (AT4G27410), *AtNAC002* (AT1G01720), *AtNAC081* (AT5G08790), *AtNAC102* (AT5G63790), *AtNAC032* (AT1G77450), *AtSID2* (AT1G74710), *AtP5CS1* (AT2G39800), *AtABI5* (AT2G36270), *AtGRP4* (AT3G23830), *AtWRKY48* (AT3G01970), *AtSnRK2.8* (AT1G78290), *AtLUP1* (AT1G78970), *AtHASPIN* (AT1G09450), *AtMCM5* (AT2G07690), *AtMYB48* (AT3G46130), *AtNUDT7* (AT4G12720), *AtBGLU47* (AT4G21760), *AtGA3OX1* (AT1G15550), *AlNAC019* (AL1G61210), *AlACTIN2* (AL3G32160), *AlNAC032* (AL2G37650), *AlNAC072* (AL7G25380), *Hpa ITS-5.8S* (EU049263.1).

SI References

1. Cao H, Glazebrook J, Clarke JD, Volko S, & Dong XN (1997) The Arabidopsis NPR1 gene that controls systemic acquired resistance encodes a novel protein containing ankyrin repeats. *Cell* 88(1):57-63.
2. Wu G & Poethig RS (2006) Temporal regulation of shoot development in Arabidopsis thaliana by miR156 and its target SPL3. *Development* 133(18):3539-3547.
3. Nobuta K*, et al.* (2007) The GH3 acyl adenylase family member PBS3 regulates salicylic acid-dependent defense responses in Arabidopsis. *Plant physiology* 144(2):1144-1156.
4. Takasaki H*, et al.* (2015) SNAC-As, stress-responsive NAC transcription factors, mediate ABA-inducible leaf senescence. *The Plant Journal* 84(6):1114-1123.
5. Yoshida T*, et al.* (2010) AREB1, AREB2, and ABF3 are master transcription factors that cooperatively regulate ABRE-dependent ABA signaling involved in drought stress tolerance and require ABA for full activation. *The Plant Journal* 61(4):672-685.
6. Adie BAT*, et al.* (2007) ABA Is an Essential Signal for Plant Resistance to Pathogens Affecting JA Biosynthesis and the Activation of Defenses in Arabidopsis. *The Plant Cell* 19(5):1665-1681.
7. Wildermuth MC, Dewdney J, Wu G, & Ausubel FM (2001) Isochorismate synthase is required to synthesize salicylic acid for plant defence. *Nature* 414(6863):562-565.
8. Hu TT*, et al.* (2011) The Arabidopsis lyrata genome sequence and the basis of rapid genome size change. *Nat Genet* 43(5):476-481.
9. Tsuda K, Sato M, Stoddard T, Glazebrook J, & Katagiri F (2009) Network Properties of Robust Immunity in Plants. *Plos Genetics* 5(12).
10. Feys BJ*, et al.* (2005) Arabidopsis SENESCENCE-ASSOCIATED GENE101 Stabilizes and Signals within an ENHANCED DISEASE SUSCEPTIBILITY1 Complex in Plant Innate Immunity. *The Plant Cell* 17(9):2601-2613.
11. Ruhe J*, et al.* (2016) Obligate Biotroph Pathogens of the Genus Albugo Are Better Adapted to Active Host Defense Compared to Niche Competitors. *Front Plant Sci* 7:820.
12. Katagiri F, Thilmony R, & He SY (2002) The Arabidopsis thaliana-pseudomonas syringae interaction. *The Arabidopsis book / American Society of Plant Biologists* 1:e0039.
13. Mine A*, et al.* (2017) An incoherent feed-forward loop mediates robustness and tunability in a plant immune network. *EMBO reports* 18(3):464-476.
14. Anders S, Pyl PT, & Huber W (2015) HTSeq—a Python framework to work with high-throughput sequencing data. *Bioinformatics* 31(2):166-169.
15. Robinson MD, McCarthy DJ, & Smyth GK (2010) edgeR: a Bioconductor package for differential expression analysis of digital gene expression data. *Bioinformatics* 26(1):139-140.
16. Robinson MD & Oshlack A (2010) A scaling normalization method for differential expression analysis of RNA-seq data. *Genome biology* 11(3):R25.
17. Ritchie ME*, et al.* (2015) limma powers differential expression analyses for RNA-sequencing and microarray studies. *Nucleic acids research* 43(7):e47.
18. JDSwcfAJ B, Dabney A, & Robinson D (2015) qvalue: Q-value estimation for false discovery rate control. *R package version 2.5.2, http://github.com/jdstorey/qvalue*.
19. Saldanha AJ (2004) Java Treeview-extensible visualization of microarray data. *Bioinformatics* 20(17):3246-3248.
20. Villajuana-Bonequi M*, et al.* (2014) Elevated salicylic acid levels conferred by increased expression of ISOCHORISMATE SYNTHASE 1 contribute to hyperaccumulation of SUMO1 conjugates in the Arabidopsis mutant early in short days 4. *The Plant journal : for cell and molecular biology* 79(2):206-219.
21. Pitzschke A, Fraundorfer A, Guggemos M, & Fuchs N (2015) Antioxidative responses during germination in quinoa grown in vitamin B-rich medium. *Food Science & Nutrition* 3(3):242-251.
22. Ziegler J*, et al.* (2014) Simultaneous analysis of apolar phytohormones and 1-aminocyclopropan-1-carboxylic acid by high performance liquid chromatography/electrospray negative ion tandem mass spectrometry via 9-fluorenylmethoxycarbonyl chloride derivatization. *J Chromatogr A* 1362:102-109.
23. Bai Y*, et al.* (2015) Functional overlap of the Arabidopsis leaf and root microbiota. *Nature* 528(7582):364-369.
24. Zgadzaj R*, et al.* (2016) Root nodule symbiosis in Lotus japonicus drives the establishment of distinctive rhizosphere, root, and nodule bacterial communities. *Proceedings of the National Academy of Sciences* 113(49):E7996-E8005.
25. Wickham H (2009) *ggplot2: Elegant Graphics for Data Analysis* (Springer-Verlag New York).

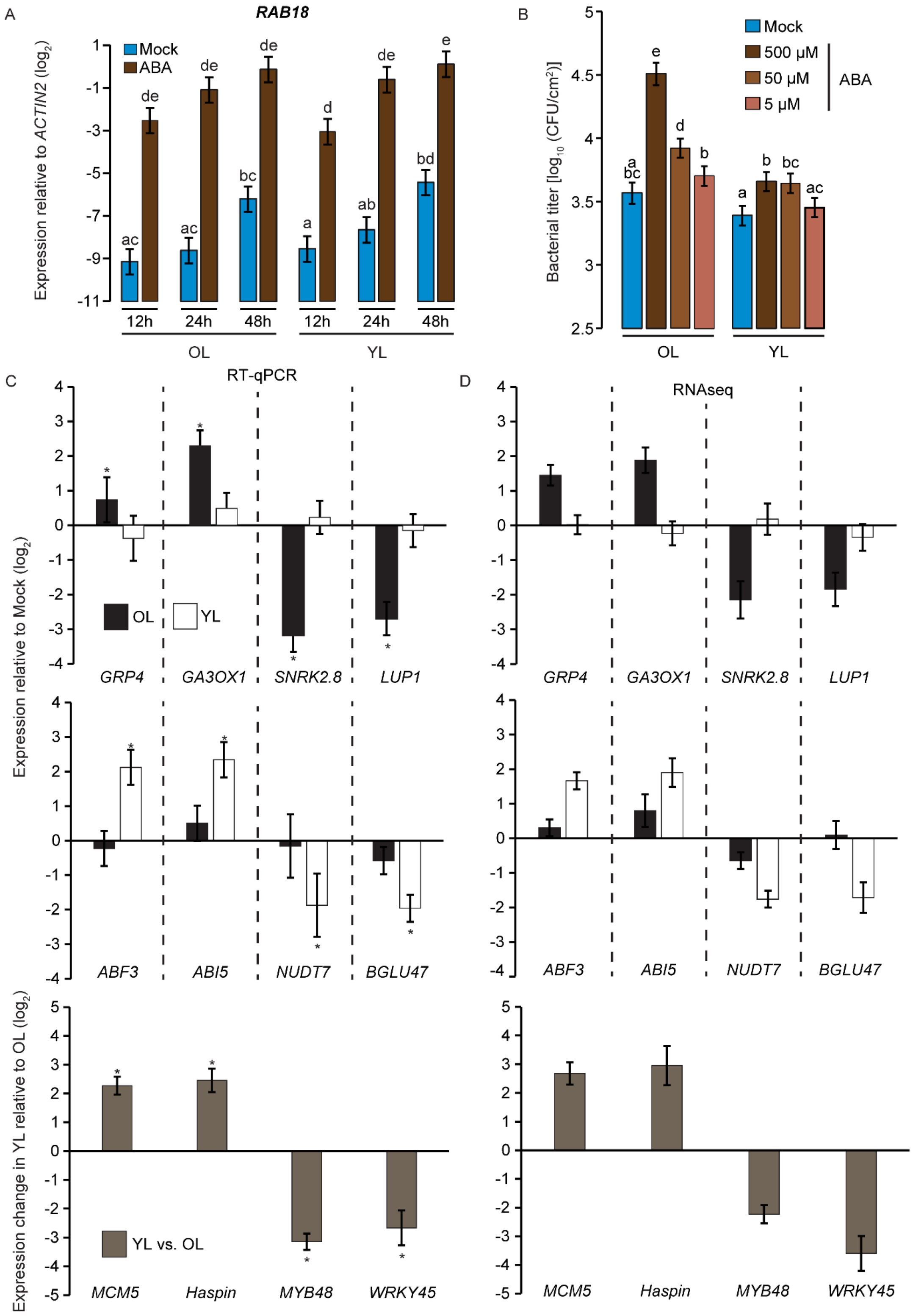

Fig. S1. ABA influences a subset of responses in a leaf age-dependent manner. (A) *RAB18* expression levels in OL and YL of Col-0 plants treated with 500 µM ABA or mock at the indicated time points. Data represent means ± SEM of at least three biological replicates using a mixed linear model. Different letters indicate significant differences (adjusted $P < 0.05$). (B) Bacterial growth after ABA treatment. OL and YL of 4-5 week-old Col-0 plants were infiltrated with *Pto* DC3000 *hrcC* (OD_{600} = 0.0002) 24 h after ABA (500, 50, or 5 µM) spray or mock treatment. Bacterial growth was measured at 2 dpi. Data represent means ± SEM of two independent experiments each with at least five biological replicates using a mixed linear model. Different letters indicate significant differences (adjusted $P < 0.005$). (C and D) The expression changes in L7 and L12 of 4-5 week-old Col-0 plants 48 h after 500 µM ABA spray compared to mock (top and middle panels) or the expression changes in L12 compared to L7 of 4-5 week-old Col-0 plants 48 h after mock treatment (bottom panels). Expression was measured by RT-qPCR (C) or represents data from the RNAseq (D). Data represent means ± SEM of at least three biological replicates. *, $P < 0.05$; **, $P < 0.01$; two-tailed Student's t-tests.

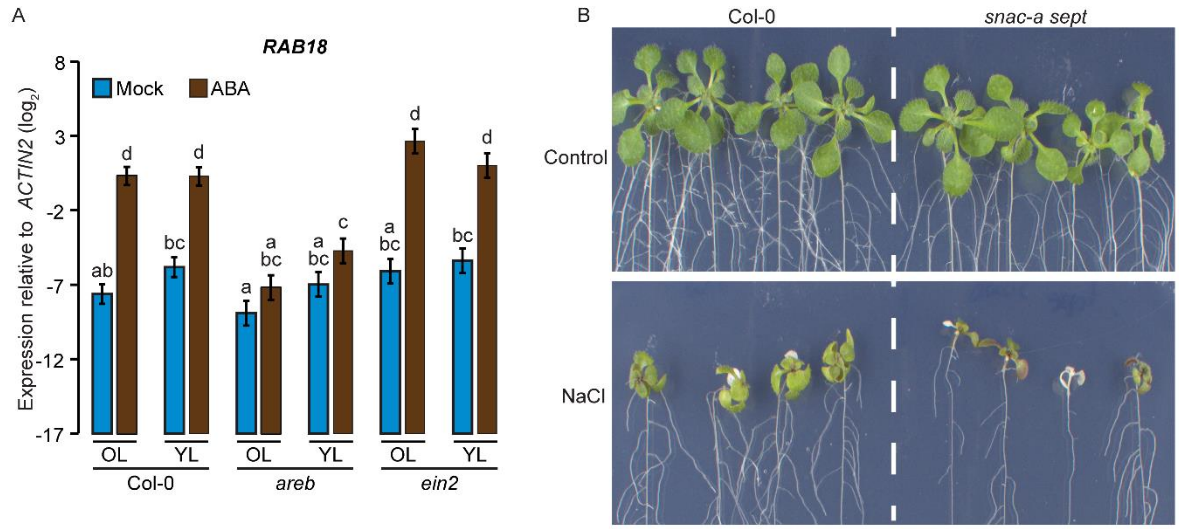

Fig. S2. The s*nac-a septuple* mutant exhibits reduced tolerance to salt stress. (A) *RAB18* expression levels in OL and YL of 4-5 week-old Col-0, *areb*, and *ein2* plants 24 h after 500 µM ABA spray or mock treatment. Data are means ± SEM of at least three biological replicates using a mixed linear model. Different letters indicate significant differences (adjusted P < 0.05) (B) Growth phenotype of Col-0 and *snac-a septuple* mutant (*snac-a sept*) seedlings 10 days after 100 mM NaCl or control treatment.

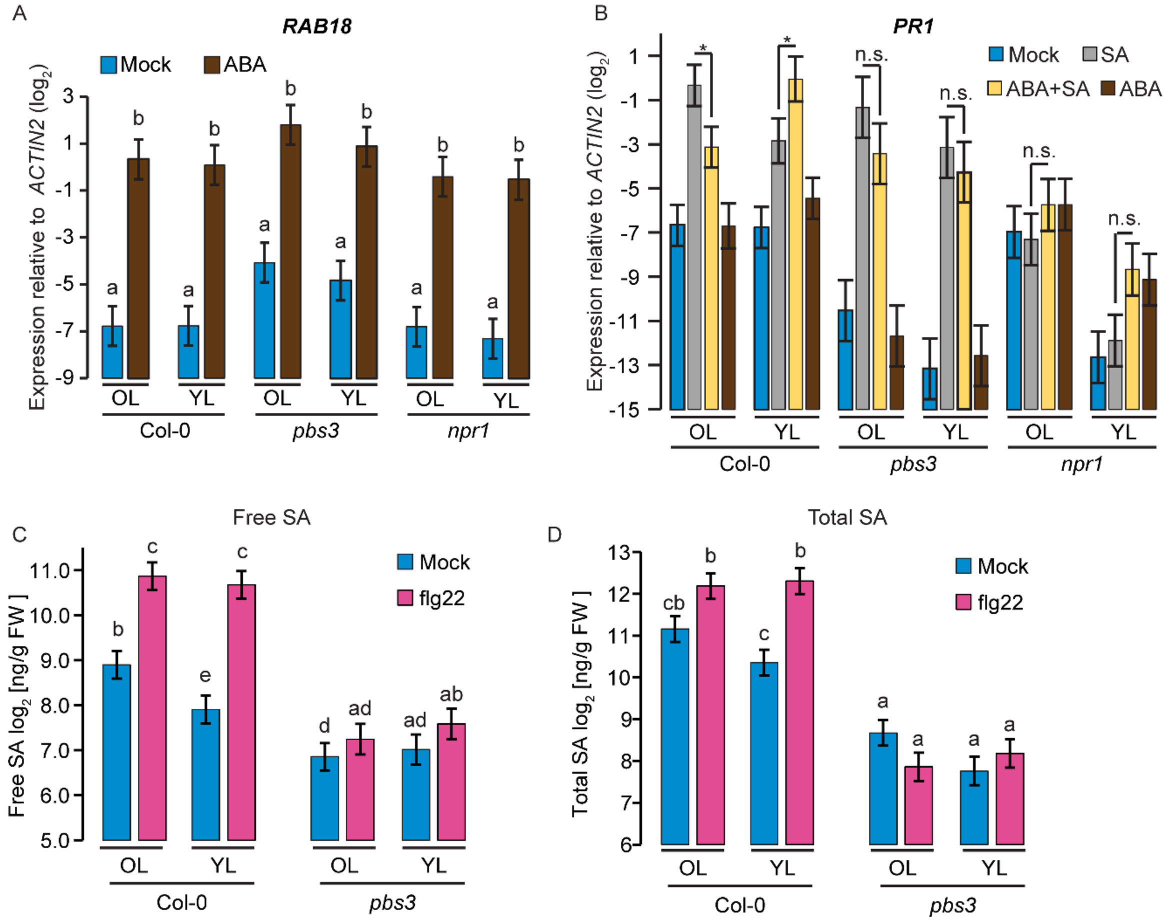

Fig. S3. Leaf age-dependent variation in different plant genotypes. (A and B) *RAB18* (A) and *PR1* (B) expression levels in OL and YL of 4-5 week-old Col-0, *pbs3,* and *npr1* plants 48 h after 500 µM ABA spray or mock treatment (A) or 24 h after 500 µM SA spray or mock treatment following 500 µM ABA spray pretreatment or mock treatment for 24 h (B). Data are means ± SEM of at least three biological replicates using a mixed linear model. (A) Different letters indicate significant differences (adjusted $P < 0.05$). (B) *, $P < 0.05$; two-tailed Student's t-tests. n.s., not significant. (C and D) Free (C) and total SA (D) amounts in OL and YL of 4-5 week-old Col-0 and *pbs3* plants 9 h after treatment with 10 µM flg22 or mock. Data represent means ± SEM of at least three biological replicates using a mixed linear model. Different letters indicate significant differences (adjusted $P < 0.05$).

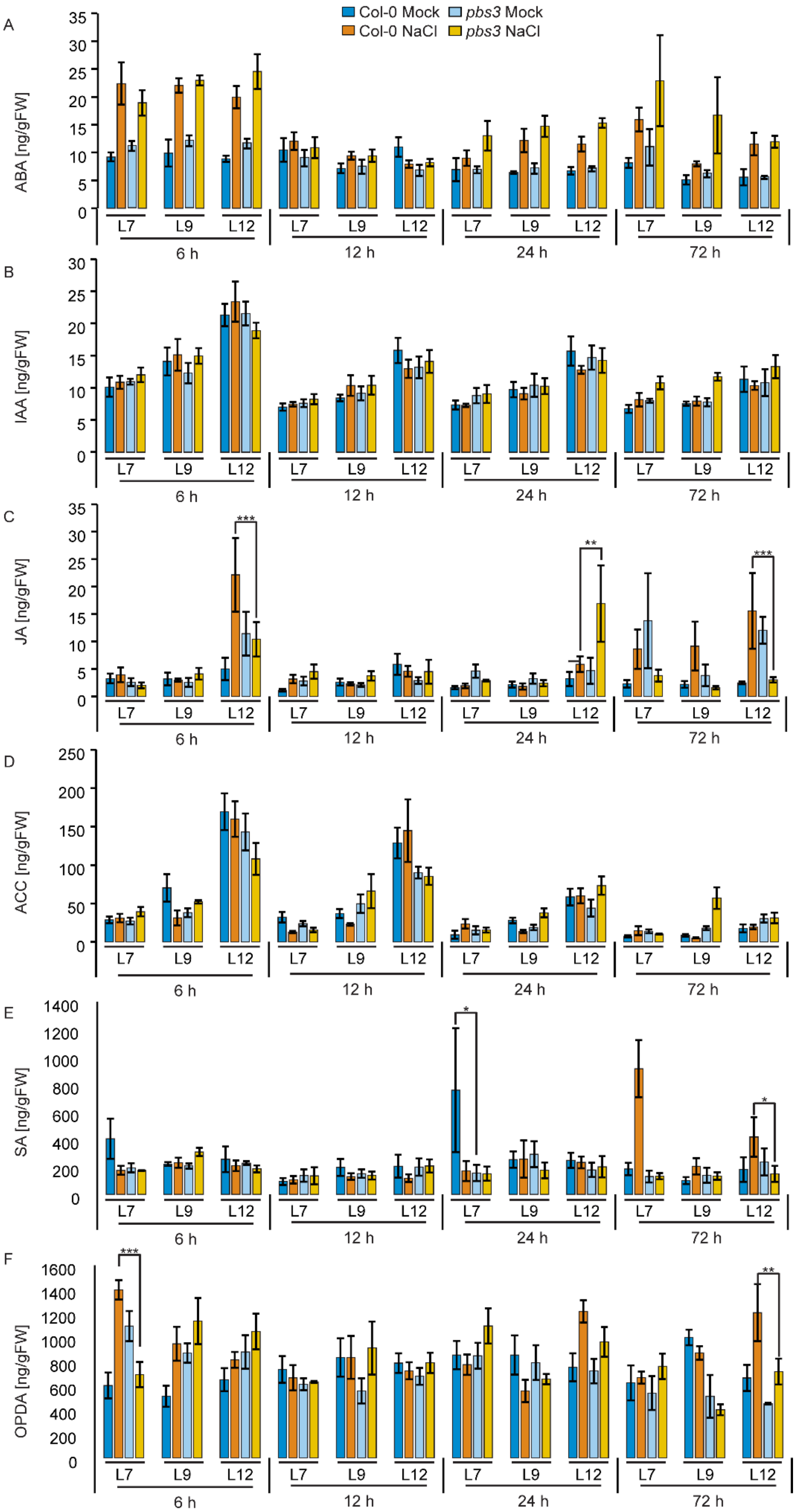

Fig. S4. Leaf-age specific accumulation of phytohormones. The amounts of ABA (A), IAA (B), JA (C), ACC (D), SA (E), and OPDA (F) in OL (L7), ML (L9), and YL (L12) of 4-5 week-old Col-0 and *pbs3* plants 6, 12, 24, and 72 h after 100 mM NaCl or mock soil drench treatment. Data represent means ± SEM of at least three biological replicates. ***, $P < 0.005$; **, $P < 0.01$; *, $P < 0.05$; two-tailed Student's t-test.

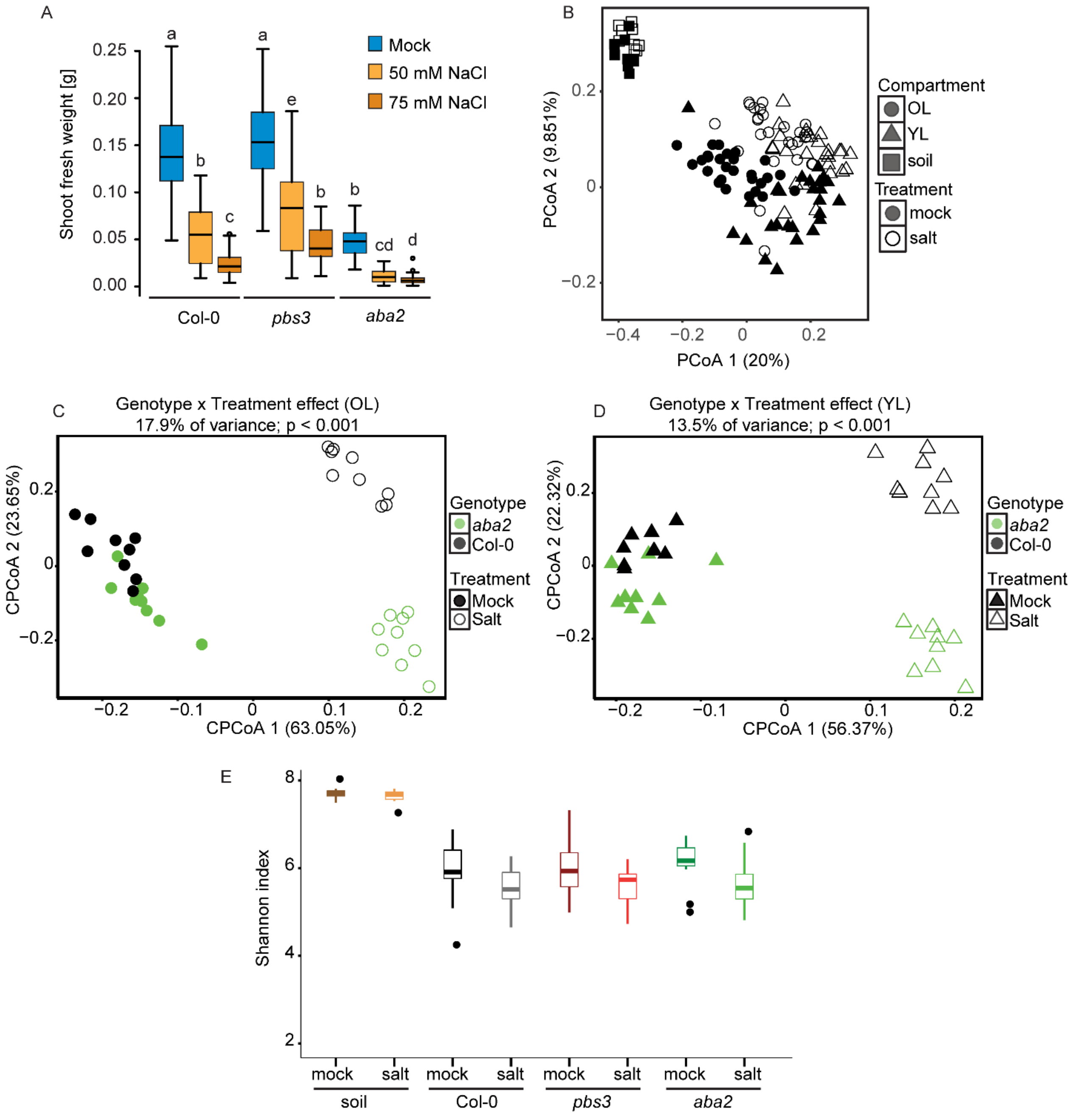

Fig. S5. Phytohormone signaling affects bacterial community composition. (A) Shoot fresh weight of Col-0, *pbs3*, and *aba2* plants grown in natural Cologne soil treated with 50 mM NaCl, 75 mM NaCl, or water (mock) for 6 weeks. The box plots show combined data from three independent experiments each with at least 10 biological replicates. Different letters indicate significant differences (adjusted $P < 0.05$). (B to D) Plants were grown in natural Cologne soil treated with 75 mM NaCl or water (mock) for 6 weeks. (B) Canonical analysis of principle coordinates of Bray-Curtis distances of bacterial beta diversity based on bacterial 16S rRNA profiling of soil and leaf communities in Col-0, *pbs3*, and *aba2* plants. (C and D) Constrained PCoA plots of Bray-Curtis distances of bacterial beta diversity based on bacterial 16S rRNA profiling of leaf bacterial communities in wild type Col-0 and *aba2* plants. Constrained analysis was performed for Genotype x Treatment effect in OL (C) or YL (D). (E) Comparison of the Shannon diversity index between soil and plant leaves. OL, old leaves and YL, young leaves.

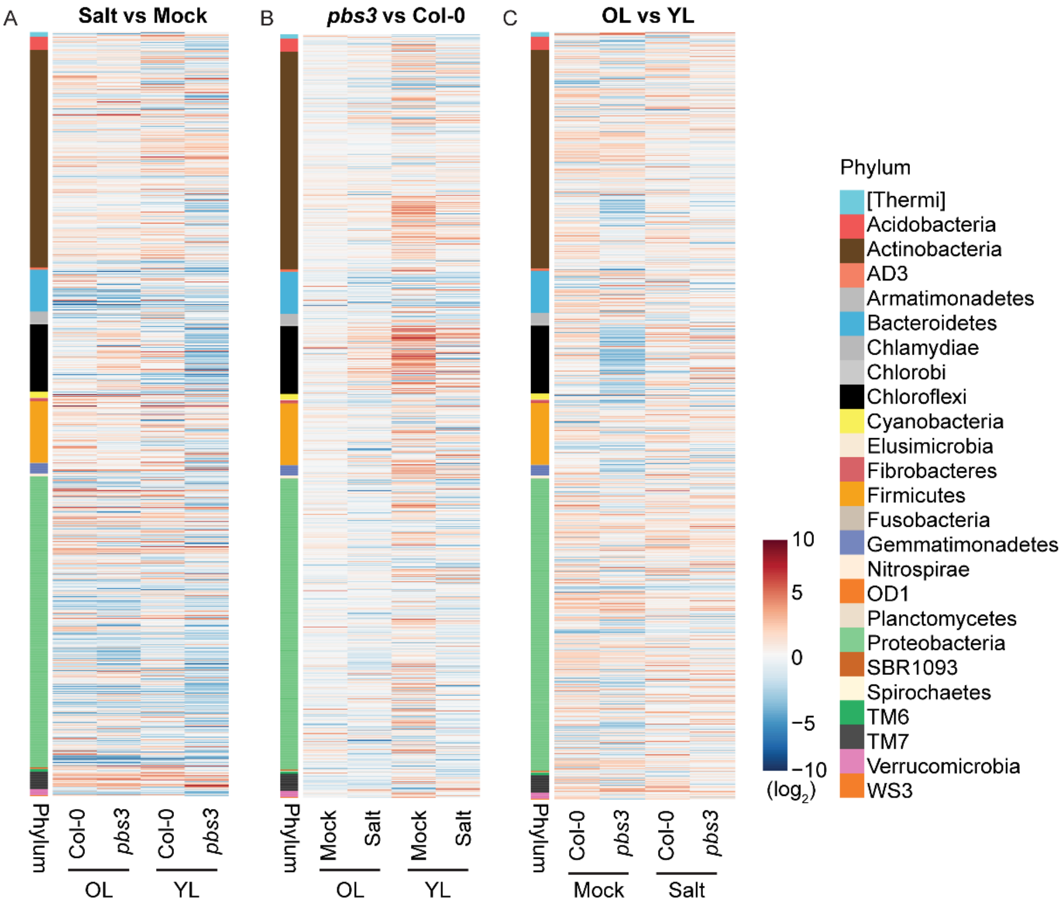

Fig. S6. *PBS3*, salt stress, and leaf age affect relative bacterial abundance. (A) Heatmap displaying $\log_2$ fold changes of relative abundance for bacterial OTUs under salt stress compared to mock in OL and YL of wild type Col-0 and *pbs3* plants. (B) Heatmap displaying $\log_2$ fold changes of relative abundance for bacterial OTUs in OL and YL of *pbs3* compared to Col-0 plants under mock or salt conditions. (C) Heatmap displaying $\log_2$ fold changes of relative abundance for bacterial OTUs in OL compared to YL of Col-0 and *pbs3* plants under mock or salt conditions. (A to C) Plants were grown in natural Cologne soil treated with water (Mock) or 75 mM NaCl (Salt) for six weeks. OL, old leaves and YL, young leaves. The phylum that each OTU belongs to is indicated by the colored bar.
